# Dataset Growth in Medical Image Analysis Research

**DOI:** 10.3390/jimaging7080155

**Published:** 2021-08-20

**Authors:** Nahum Kiryati, Yuval Landau

**Affiliations:** School of Electrical Engineering, Tel Aviv University, Tel Aviv 69978, Israel; yuvi.landa7@gmail.com

**Keywords:** dataset size, human subjects, medical image analysis, MICCAI conferences

## Abstract

Medical image analysis research requires medical image datasets. Nevertheless, due to various impediments, researchers have been described as “data starved”. We hypothesize that implicit evolving community standards require researchers to use ever-growing datasets. In Phase I of this research, we scanned the MICCAI (Medical Image Computing and Computer-Assisted Intervention) conference proceedings from 2011 to 2018. We identified 907 papers involving human MRI, CT or fMRI datasets and extracted their sizes. The median dataset size had grown by 3–10 times from 2011 to 2018, depending on imaging modality. Statistical analysis revealed exponential growth of the geometric mean dataset size with an annual growth of 21% for MRI, 24% for CT and 31% for fMRI. Thereupon, we had issued a forecast for dataset sizes in MICCAI 2019 well before the conference. In Phase II of this research, we examined the MICCAI 2019 proceedings and analyzed 308 relevant papers. The MICCAI 2019 statistics compare well with the forecast. The revised annual growth rates of the geometric mean dataset size are 27% for MRI, 30% for CT and 32% for fMRI. We predict the respective dataset sizes in the MICCAI 2020 conference (that we have not yet analyzed) and the future MICCAI 2021 conference.

## 1. Introduction

Medical image analysis is an active research field focusing on computational methods for the extraction of clinically useful information from medical images. Research in medical image analysis critically depends on the availability of relevant medical image sets (datasets) for tasks, such as training, testing and validation of algorithms. It is widely accepted that solid medical image analysis research requires the use of sufficiently large datasets. This notion is rooted in classical statistical estimation [1] and classification [2] theories and is supported by the state-of-the-art machine learning theory [3].

Obtaining relevant medical images is challenging and costly, as it requires the cooperation of medical professionals and institutes and alleviation of ethical, legal and often commercial conflicts. For example, making a clinical medical image available for research usually requires a collaborating clinical expert to obtain regulatory approvals, to find a relevant image in an institutional archive, to interpret the image and to remove identifying details. Thus, for research in medical image analysis, relevant images are usually in high demand and short supply. In a recent conference [4], “the common theme from attendees was that everyone participating in medical image evaluation with machine learning is data starved”. Thus, typical dataset sizes in medical image analysis research are a far cry from the common perception of big data in healthcare [5].

Since 2012, the deep learning paradigm has revolutionized the field of medical image analysis [6], underscoring the significance of large image datasets. Thus, researchers are torn between the need for large image datasets, i.e., datasets containing medical images of many subjects, and the cost and effort required to obtain them.

In contrast to classical estimation, detection and classification problems, where sample size planning may yield to analysis [1,2,7,8,9,10,11], the theoretical understanding of deep learning is limited. The trend is that increasing the training dataset size improves the performance of deep learning networks [3,12]. Small datasets are associated with overfitting and poor generalization performance on unseen data [12]. In the presence of rare pathologies, small datasets are known to result in class imbalance and inadequate training [4]. Yet, solid objective criteria for dataset size are difficult to obtain, especially in medical image analysis.

In practice, since dataset size is associated with research quality, the research community develops expectations regarding dataset size in medical image analysis. These evolving expectations are reflected in the peer-review process of reputable publication venues, implicitly setting ad-hoc thresholds on dataset size. Thus, a manuscript is accepted for publication or presentation in a reputable venue only if the dataset size is regarded by the referees as sufficiently large. Consequently, dataset sizes appearing in published articles reflect the implicit standard of the research community for dataset size. Since the standard is not static, researchers are often dismayed to discover that the dataset used in their current study, where the number of subjects was previously considered sufficiently large, is no longer up to expectations. This uncertainty leads to marginalization and loss of potentially valuable research.

The purpose of this research is to better understand the temporal trends in the implicit community expectations regarding dataset size. We hypothesize that these expectations grow over time, such that ever-increasing datasets are required for acceptance of a manuscript to a reputable venue. This study provides researchers, funding agencies, program committees and editors with guidelines regarding the community standards for dataset size in current and future medical image analysis research. For example, when preparing a research proposal for a funding agency, our results will allow the investigator to plan the dataset size and its evolution over the research period and to present a convincing justification for the requested dataset cost.

## 2. Methods

We scanned the proceedings of the annual MICCAI conference from 2011 to 2019 [13,14,15,16,17,18,19,20,21], and carefully extracted the numbers of human subjects included in the datasets used. MICCAI, the acronym for Medical Image Computing and Computer-Assisted Intervention, is a leading conference in the field, with a rigorous peer-review process (typically at least three reviewers, double-blind). Table 1 shows the number of submitted papers, the number of accepted papers (oral and posters) and the acceptance ratio per year (main conference only, excluding satellite events). We preferred monitoring a conference series rather than an archival journal since the conference review period is shorter than that of quality journals, implying a shorter sampling aperture, hence better temporal sampling.

We focused on studies involving three important imaging modalities: Magnetic Resonance Imaging (MRI), Computed Tomography (CT) and Functional MRI (fMRI). Taken together, this selection covers a substantial portion of the research articles in the MICCAI proceedings. Being well-established modalities, a good number of articles associated with each modality is found in each annual edition of the MICCAI proceedings, allowing meaningful statistical study.

We only considered datasets referring to human subjects rather than animal or other datasets. Given the stringent regulatory framework regarding human data [22], we believe that the challenges and trade-offs associated with the collection and use of human medical data are unique and justify the exclusion of non-human datasets from this study. Nevertheless, in utero and post-mortem human datasets are included. We define the dataset size to be the number of distinct human subjects rather than the number of test images or similar data structures, as the number of human subjects better reflects the recruitment effort.

We examined each of the 2676 articles in the MICCAI 2011–2019 proceedings, found the 1215 relevant articles and extracted from each article the dataset size. No distinction was made between data used for training, validation, testing or any other use. With few exceptions, articles associated with several imaging modalities were considered with regard to one of the modalities, with preference to fMRI over CT and MRI. Table 2 shows the number of relevant MICCAI articles, i.e., the articles included in our analysis, per year and imaging modality. The growth in 2018 and 2019 corresponds to the larger overall number of articles in the respective MICCAI conferences (see Table 1).

To obtain an initial overview of dataset sizes in the MICCAI 2011–2019 conferences (Section 3), for each year and modality, we present the average, geometric mean and median dataset sizes. In computing the average, to reduce the effect of outliers, we discarded the largest value and smallest value.

The statistical analysis (Section 4 and Section 5) had been carried out in two phases. Phase I (Section 4) had been carried out before MICCAI 2019 took place. Statistical analysis of the 2011–2018 data was performed for each modality separately, using SPSS v.25 (IBM, Armonk NY, USA) and R v.3.6.0 25 (R-Foundation, Vienna, Austria). *p*-values were corrected for multiple comparisons using the Benjamini–Hochberg (BH) procedure, with *p* < 0.05 considered as significant. Phase I culminated in the release of predictions [23] regarding dataset sizes in the then-upcoming MICCAI 2019 conference [21]. 

In Phase II (Section 5), the MICCAI 2019 statistics were extracted and compared to the corresponding Phase I predictions. The statistical analysis was then revised to include the 2019 data and cover the entire 2011–2019 period. Based on this analysis, we issue predictions regarding dataset sizes in the MICCAI 2020 conference (its analysis is beyond the scope of this research) and the MICCAI 2021 conference, noting that it has not yet taken place at the time of writing.

## 3. Descriptive Results

**Figure 1:**
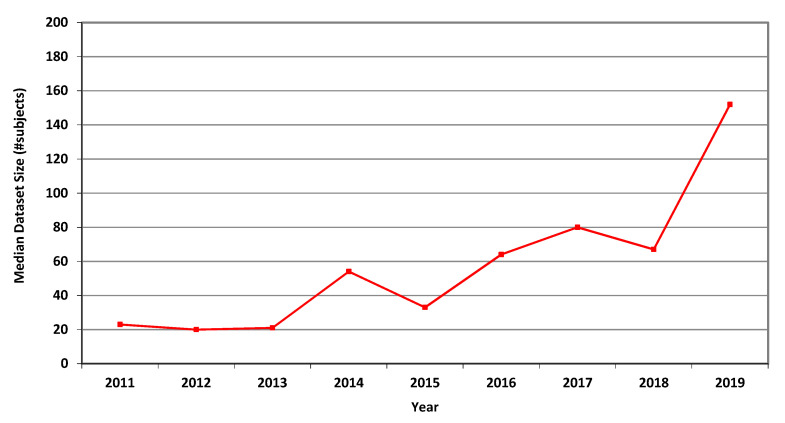
Median size of datasets used in MICCAI articles related to MRI in each of the years 2011–2019.

For MRI, the annual average, geometric mean and median dataset sizes used in MICCAI articles in each of the years 2011–2019 are shown in Table 3. It can be seen that all three measures greatly increased over that period. The higher values of the average values follow from the occasional use of large publicly accessible datasets, see [24], affecting the averages but not the median values. A graphical representation of the median values per year is shown in Figure 1.

**Figure 2:**
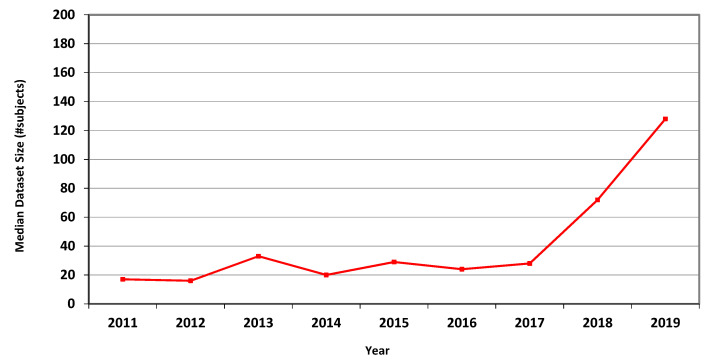
Median size of datasets used in MICCAI articles related to CT in each of the years 2011–2019.

**Figure 3:**
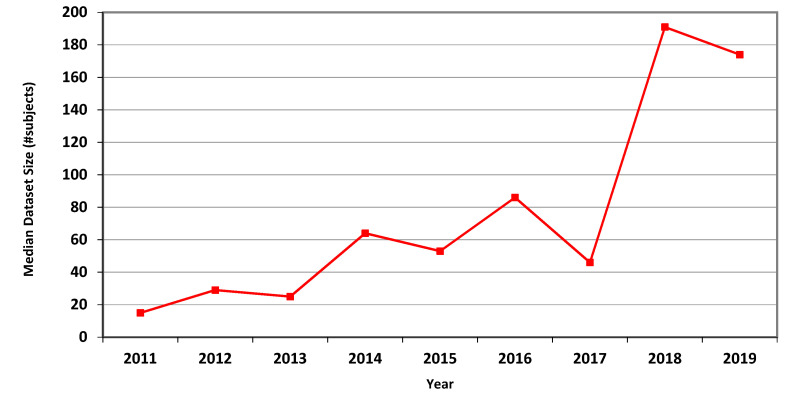
Median size of datasets used in MICCAI articles related to fMRI in each of the years 2011–2019.

The measures for CT are presented in Table 4, showing marked overall dataset growth over the study period. For CT, the graph of median dataset size per year is shown in Figure 2. The corresponding data for fMRI is presented in Table 5 and in Figure 3.

## 4. Statistical Analysis and Prediction—Phase I (2011–2018 Data)

As a preliminary test of the dataset growth hypothesis, we first divided the range of years into two categories: 2011–2014 and 2015–2018. The distributions of the dataset sizes in the two categories were similar and non-normal. A Mann–Whitney U test was carried out to determine if there were differences in dataset sizes between the two time-categories.

- For MRI, the median numbers of subjects for 2011–2014 (26) and 2015–2018 (55.5) were statistically significantly different, U = 19.115, *p* < 0.001.
- For CT, the median numbers of subjects for 2011–2014 (18.5) and 2015–2018 (36) were statistically significantly different, U = 8.311, *p* = 0.006.
- For fMRI, the median numbers of subjects for 2011–2014 (37) and 2015–2018 (77) were statistically significantly different, U = 10.493, *p* = 0.003.

These results corroborate the dataset-growth hypothesis.

Returning to the full Phase-I year range, 2011–2018, we used the natural logarithm (ln) transformation to normalize dataset sizes. Linear regression established that the year (after 2010) could statistically significantly predict the natural logarithm of the dataset size. Throughout this research, in each regression analysis, the regression is based on the whole ensemble of dataset sizes, not on the empirical geometric means. The predictions for 2019 are of special interest since MICCAI 2019 had not yet taken place at the time these predictions were made.

For MRI, the model was statistically significant, F(1,566) = 38.720, *p* < 0.001. The model explained 6.2% (adjusted R^2^) of the variance in the natural logarithm of dataset sizes. The year was statistically significant (B = 0.189, CI = (0.129, 0.249), *p* < 0.001), where B denotes slope and CI is its confidence interval. The regression equation is

(1) E^(lnN)=2.771+0.189(y−2010)

where Ê(ln N) is the predicted mean of the natural logarithm of dataset sizes and y is the year. Returning to the original scale of MRI dataset sizes, we obtain

(2) G^(N)=e2.771+0.189(y−2010)

**Figure 4:**
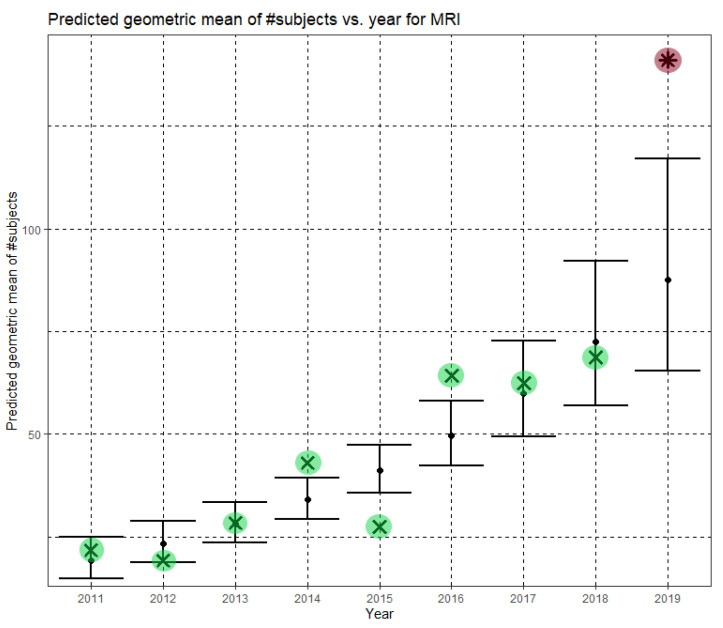
Phase I—Predicted geometric mean (black dots) and confidence intervals of dataset sizes in MICCAI articles involving MRI for the years 2011–2019, based on the whole ensemble of 2011–2018 MRI dataset sizes. The empirical geometric means (2011–2018) are shown (in green) for comparison (x marks). The empirical geometric mean for 2019 (in purple) became available later, in Phase II of this research.

Here, Ĝ(N) is the predicted geometric mean of MRI dataset sizes. The annual growth rate of the predicted geometric mean is about 21%, corresponding to (e^0.189^ − 1). Figure 4 shows Ĝ(N) with its confidence interval for each of the years 2011–2019. The empirical geometric means, taken from Table 3, are shown (in green) for comparison. We predicted the geometric mean of MRI dataset sizes in MICCAI 2019 to be 87.5, with a confidence interval of (65.5, 116.9). The empirical geometric mean for 2019 (in purple) became available later, in Phase II of this research.

The regression model was statistically significant for CT as well, F(1,221) = 21.273, *p* < 0.001. The model explained 8.4% (adjusted R^2^) of the variance in the natural logarithm of the dataset sizes. The year was statistically significant (B = 0.213, (CI = 0.122, 0.305), *p* < 0.001). The regression equation is

(3) E^(lnN)=2.456+0.213(y−2010)

Returning to the original scale of CT dataset sizes, we obtain

(4) G^(N)=e2.456+0.213(y−2010)

**Figure 5:**
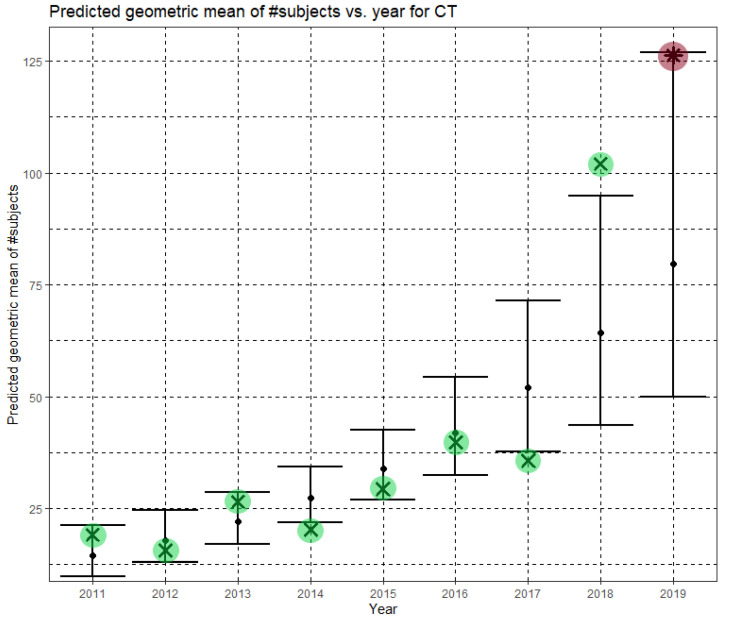
Phase I—Predicted geometric mean (black dots) and confidence intervals of dataset sizes in MICCAI articles involving CT for the years 2011–2019, based on the whole ensemble of 2011–2018 CT dataset sizes. The empirical geometric means (2011–2018) are shown (in green) for comparison (x marks). The empirical geometric mean for 2019 (in purple) became available later, in Phase II of this research.

In Equation (4), Ĝ(N) is the predicted geometric mean of CT dataset sizes. Here, the annual growth rate of the predicted geometric mean is about 24%. Figure 5 shows Ĝ(N) with its confidence interval for each of the years 2011–2019. The empirical geometric means, taken from Table 4, are shown (in green) for comparison. We predicted the geometric mean of CT dataset sizes in the MICCAI 2019 conference to be 79.6 with a confidence interval of (49.9, 126.9). The empirical geometric mean for 2019 (in purple) became available later, in Phase II of this research.

Finally, for fMRI, the model was yet again statistically significant, F(1,114) = 27.130, *p* < 0.001. The model explained 18.5% (adjusted R^2^) of the variance in the natural logarithm of dataset sizes. The year was statistically significant (B = 0.271, CI = (0.168,0.374), *p* < 0.001). The regression equation is

(5) E^(lnN)=2.683+0.271(y−2010)

Returning to the original scale of fMRI dataset sizes, we obtain

(6) G^(N)=e2.683+0.271(y−2010)

**Figure 6:**
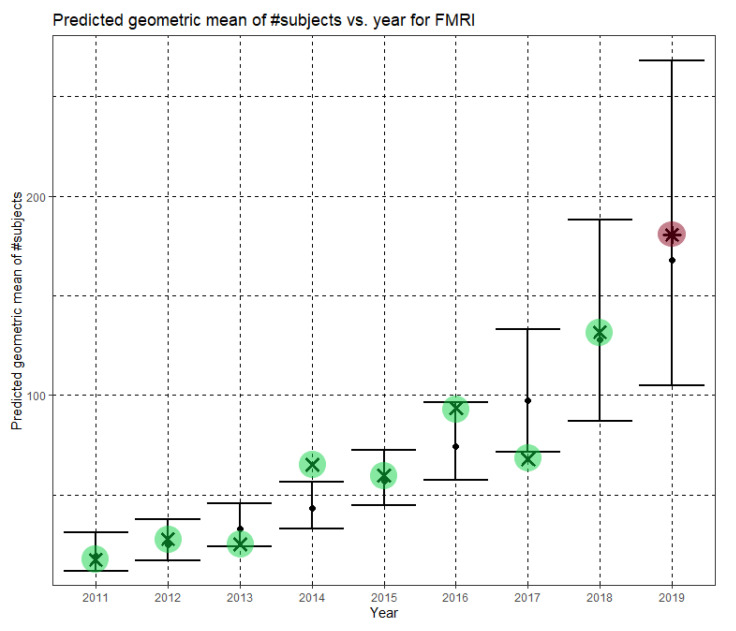
Phase I—Predicted geometric mean (black dots) and confidence intervals of dataset sizes in MICCAI articles involving fMRI for the years 2011–2019, based on the whole ensemble of 2011–2018 fMRI dataset sizes. The empirical geometric means (2011–2018) are shown (in green) for comparison (x marks). The empirical geometric mean for 2019 (in purple) became available later, in Phase II of this research.

In Equation (6), Ĝ(N) is the predicted geometric mean of fMRI dataset sizes. For fMRI, the annual growth rate of the predicted geometric mean is about 31%. Figure 6 shows Ĝ(N) with its confidence interval for each of the years 2011–2019. The empirical geometric means, taken from Table 5, are shown (in green) for comparison. We predicted the geometric mean of fMRI dataset sizes in the MICCAI 2019 conference to be 167.7, with a confidence interval of (104.9, 268.0). The empirical geometric mean for 2019 (in purple) became available later, in Phase II of this research.

The low adjusted R^2^ values in this section (6.2% for MRI, 8.4% for CT and 18.5% for fMRI) and in the next section require clarification. The regression tasks in this research are unusual because for each imaging modality and for each value of the independent variable (year), there are many (10–187, see Table 2) disparate values of the dependent variable (dataset size). The values themselves are radically different from each other, as dataset sizes encountered in MICCAI articles can be as small as one or as large as many thousands (where large external datasets are used). This implies a huge inherent variance of the dependent variable at each value of the independent variable. No single-valued hypothetic regression function, regardless of linearity or of any other property, can provide a single prediction at a specific value of the independent variable that simultaneously “explains” hugely different observations of the dependent variable at that point. This is the reason for the inevitably low adjusted R^2^ values. The higher adjusted R^2^ value for fMRI, compared to MRI and CT, follows from the scarcity of large external fMRI datasets, implying a smaller inherent variance that needs to be “explained”. Nevertheless, our models are statistically significant, and the predicted geometric means are pleasantly close to the empirical ones where the latter are available.

The Phase I analysis, including predictions regarding dataset sizes in MICCAI 2019, had been presented in [23], before the MICCAI 2019 conference took place.

## 5. Statistical Analysis and Prediction—Phase II (2011–2019 Data)

Once the MICCAI 2019 proceedings became available, we extracted the dataset sizes and computed the empirical geometric means of dataset sizes for MRI, CT and fMRI (see the 2019 column in Table 3, Table 4 and Table 5, respectively). The 2019 values are shown in purple in Figure 4, Figure 5 and Figure 6. For CT and fMRI, the empirical geometric means are within the confidence intervals of the Phase I analysis. For MRI, the empirical geometric mean of dataset sizes exceeds the confidence interval, indicating dataset growth beyond the Phase I prediction.

Based on the Phase II year range 2011–2019, we updated the statistical analysis. Linear regression again established that the year (after 2010) can statistically significantly predict the natural logarithm of the dataset size. Here too, the regression is based on the whole ensemble of dataset sizes, not on the empirical geometric means. The predictions for 2020–2021 are of special interest since MICCAI 2021 has not yet taken place, and the MICCAI 2020 data has not yet been analyzed at the time of writing. For MRI, the Phase II model was statistically significant, F(1,753) = 104.727, *p* < 0.001. The model explained 12.09% (adjusted R^2^) of the variance in the natural logarithm of dataset sizes. The year was statistically significant (B = 0.240, CI = (0.194,0.286), *p* < 0.001), where B denotes slope and CI is its confidence interval. The Phase II regression equation is

(7) E^(lnN)=2.596+0.240(y−2010)

where Ê(ln N) is the predicted mean of the natural logarithm of dataset sizes, and y is the year. Returning to the original scale of MRI dataset sizes, we obtain

(8) G^(N)=e2.596+0.240(y−2010)

**Figure 7:**
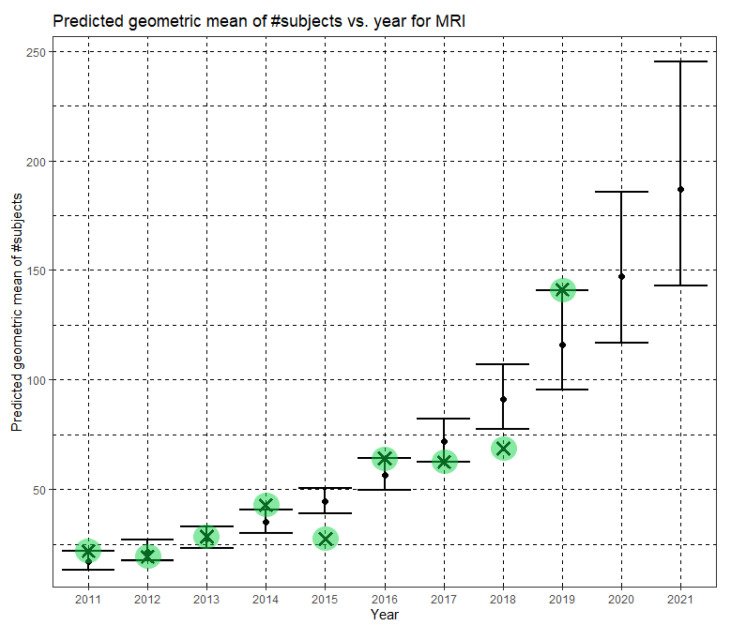
Phase II—Predicted geometric mean (black dots) and confidence intervals of dataset sizes in MICCAI articles involving MRI for the years 2011–2021 based on the whole ensemble of 2011–2019 MRI dataset sizes. The empirical geometric means (2011–2019) are shown (in green) for comparison (x marks). The empirical geometric means for 2020 and 2021 are not known at the time of writing.

Here, Ĝ(N) is the Phase II prediction of the geometric mean of MRI dataset sizes. The annual growth rate of the predicted geometric mean is about 27%, corresponding to (e^0.240^ − 1). Figure 7 shows Ĝ(N) with its confidence interval for each of the years 2011–2021. The empirical geometric means for 2011–2019, taken from Table 3, are shown (in green) for comparison. We predict the geometric mean of MRI dataset sizes in MICCAI 2020 to be 147.2, with a confidence interval of (116.9, 185.5). We predict the geometric mean of MRI dataset sizes in MICCAI 2021 to be 187.1, with a confidence interval of (142.8, 245.2). 

The Phase II regression model was statistically significant for CT as well, F(1,318) = 63.700, *p* < 0.001. The model explained 16.4% (adjusted R^2^) of the variance in the natural logarithm of dataset sizes. The year was statistically significant (B = 0.266, (CI = 0.200, 0.331), *p* < 0.001). The Phase II regression equation is

(9) E^(lnN)=2.289+0.266(y−2010)

Returning to the original scale of CT dataset sizes, we obtain

(10) G^(N)=e2.289+0.266(y−2010)

**Figure 8:**
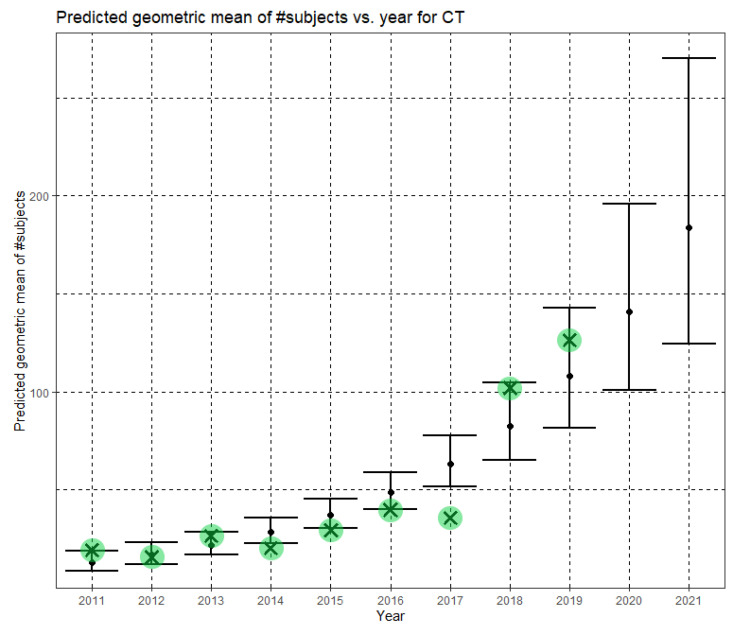
Phase II—Predicted geometric mean (black dots) and confidence intervals of dataset sizes in MICCAI articles involving CT for the years 2011–2021 based on the whole ensemble of 2011–2019 CT dataset sizes. The empirical geometric means (2011–2019) are shown (in green) for comparison (x marks). The empirical geometric means for 2020 and 2021 are not known at the time of writing.

In Equation (10), Ĝ(N) is the Phase II predicted geometric mean of CT dataset sizes. Here, the annual growth rate of the predicted geometric mean is about 30%. Figure 8 shows Ĝ(N) with its confidence interval for each of the years 2011–2021. The empirical geometric means for 2011–2019, taken from Table 4, are shown (in green) for comparison. We predict the geometric mean of CT dataset sizes in MICCAI 2020 to be 140.7, with a confidence interval of (101.1, 196.0). We predict the geometric mean of CT dataset sizes in MICCAI 2021 to be 183.6, with a confidence interval of (124.7, 270.2).

Finally, for fMRI, the Phase II model was yet again statistically significant, F(1,138) = 42.545, *p* < 0.001. The model explained 23.0% (adjusted R^2^) of the variance in the natural logarithm of dataset sizes. The year was statistically significant (B = 0.277, CI = (0.193,0.361), *p* < 0.001). The Phase II regression equation is

(11) E^(lnN)=2.661+0.277(y−2010)

Returning to the original scale of fMRI dataset sizes, we obtain

(12) G^(N)=e2.661+0.277(y−2010)

**Figure 9:**
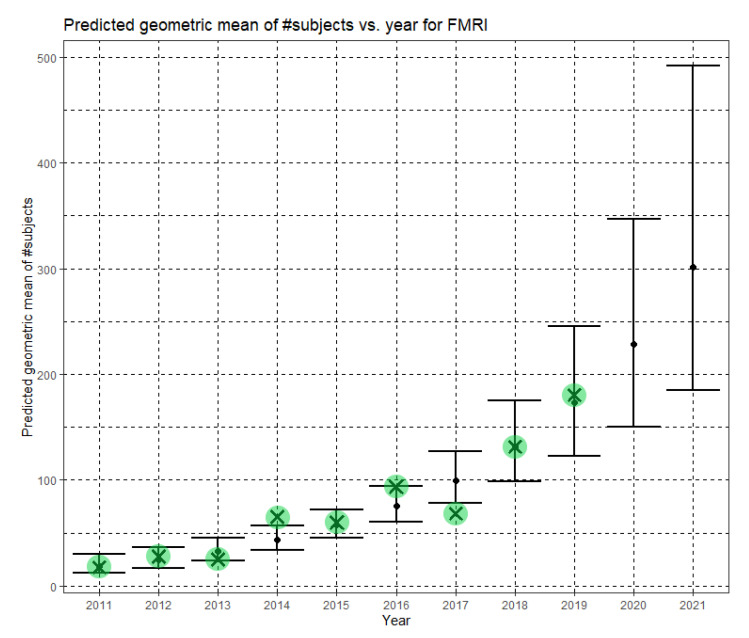
Phase II—Predicted geometric mean (black dots) and confidence intervals of dataset sizes in MICCAI articles involving fMRI for the years 2011–2021 based on the whole ensemble of 2011–2019 fMRI dataset sizes. The empirical geometric means (2011–2019) are shown (in green) for comparison (x marks). The empirical geometric means for 2020 and 2021 are not known at the time of writing.

In Equation (12), Ĝ(N) is the Phase II predicted geometric mean of fMRI dataset sizes. For fMRI, the annual growth rate of the predicted geometric mean is about 32%. Figure 9 shows Ĝ(N) with its confidence interval for each of the years 2011–2021. The empirical geometric means for 2011–2019, taken from Table 5, are shown (in green) for comparison. We predict the geometric mean of fMRI dataset sizes in MICCAI 2020 to be 228.5, with a confidence interval of (150.6, 346.6). We predict the geometric mean of fMRI dataset sizes in MICCAI 2021 to be 301.4, with a confidence interval of (184.6, 492.1).

## 6. Discussion

To our knowledge, this research is the first attempt to quantify the evolving, implicit standards of the research community regarding dataset size in medical image analysis research. Compared to common computer vision tasks, acquiring datasets for medical image analysis is difficult and expensive. For example, while annotation of the ImageNet dataset [25] was based on low-cost crowdsourcing using the Amazon Mechanical Turk, the gold standard for most medical image analysis tasks is reading by a highly qualified radiologist. Therefore, the marginal cost of enlarging a computer vision dataset is often negligible, but the cost of expanding a medical image analysis dataset can be prohibitive. Thus, prior to this research, dataset growth in medical image analysis research was not obvious, and the growth rate could not be deduced from other domains.

In Phase I of this research, we scanned all the 2136 articles published in the MICCAI proceedings from 2011 to 2018. We extracted the dataset sizes from 907 papers relying on human data from three prevalent imaging modalities: MRI, CT and fMRI. Going through so many papers required substantial effort and expertise because dataset size is often not explicitly reported. It is sometimes distributed in different parts of an article or provided as a reference to another publication. Table 3, Table 4 and Table 5 and Figure 1, Figure 2 and Figure 3 describe the data. Human dataset size in MRI-related research nearly trebled from 2011 to 2018, while the CT and fMRI datasets grew at even faster rates. Similar trends are observed in the median, geometric mean and average values, though the average numbers are substantially higher than the median and geometric mean measures due to the occasional use of very large open datasets.

Still in Phase I, statistical analysis using the Mann–Whitney U test corroborated the dataset growth hypothesis for all three modalities. Furthermore, regression analysis revealed statistically significant exponential growth in the geometric mean of the dataset sizes, albeit with large variability. The predicted annual growth rates of the geometric mean of the number of subjects in the datasets were about 21% for MRI, 24% for CT and 31% for fMRI.

For the MICCAI 2019 conference, which had not yet taken place during Phase I of this research, we predicted the geometric mean of the number of subjects in the datasets to be 87.5 with a confidence interval of (65.5, 116.9) for MRI related articles, 79.6 with a confidence interval of (49.9, 126.9) for CT and 167.7 with a confidence interval of (104.9, 268.0) for fMRI.

Phase II of this research incorporated the MICCAI 2019 data. The empirical geometric means in the MICCAI 2019 proceedings were 141.1 for MRI, 126.2 for CT and 180.5 for fMRI. For CT and fMRI, the empirical MICCAI 2019 values are within the confidence intervals of the Phase I predictions. For MRI, they are even beyond the confidence interval of the Phase I prediction. Based on analysis of the MICCAI 2019 data, we updated the regression models. The Phase II annual growth rates of the geometric mean of the number of subjects in the datasets are about 27% for MRI, 30% for CT and 32% for fMRI. In slight analogy to Moore’s law, these estimated growth rates can provide researchers, review boards and funding agencies with a tentative roadmap regarding dataset sizes in future medical image analysis studies.

This study estimated dataset growth in medical image analysis research by analyzing the annual MICCAI conference proceedings. The extension to prominent journals in the field is an interesting topic for future research. Note, however, that the review period for conferences is short, identical for all papers and synchronized by the common submission and reviewing timeline. For reputable journals, the review period is longer, different between papers and unsynchronized. These differences raise substantial analysis challenges. Comparative dataset growth analysis across different scientific domains is an additional interesting future research topic. For example, ImageNet currently contains more than 14 million images. We assume that the growth rate in a particular field depends on the data acquisition and annotation cost in that field.

The perception that “everyone participating in medical image evaluation with machine learning is data starved” [4] is not surprising given the exponentially increasing expectations regarding dataset size. Transfer learning [26] and data augmentation [27] are two popular and often successful strategies to alleviate the shortage of data, see [28]. Recently, Generative Adversarial Networks (GAN) have been used to create large sets of “fake” but credible new medical images based on a limited collection of genuine images, see [29]. If accepted by the research community, this strategy may bridge the gap between demand and supply of medical images for use in research and development.

## Figures and Tables

**Table 1:** MICCAI submission and acceptance data (main conference).

Year	Submitted Papers	Accepted Papers	Acceptance Rate
2011	819	251	30%
2012	781	252	32%
2013	798	262	33%
2014	862	253	29%
2015	810	263	32%
2016	756	228	30%
2017	800	255	32%
2018	1068	372	35%
2019	1809	540	30%

**Table 2:** Number of articles included in the analysis, per year and imaging modality.

	2011	2012	2013	2014	2015	2016	2017	2018	2019
MRI	62	63	76	63	66	69	75	94	187
CT	36	24	20	40	23	14	30	36	97
fMRI	11	10	14	10	14	16	15	26	24

**Table 3:** Average, geometric mean and median MRI dataset sizes (number of subjects) used in MICCAI articles in each of the years 2011–2019.

MRI	2011	2012	2013	2014	2015	2016	2017	2018	2019
average	74.3	52.2	79.9	139.2	65.6	163.6	178.1	250.6	650.2
geom. mean	21.7	19.2	28.5	43.0	27.4	64.2	62.5	68.6	141.1
median	23	20	21	54	33	64	80	67	152

**Table 4:** Average, geometric mean and median CT dataset sizes (number of subjects) used in MICCAI articles in each of the years 2011–2019.

CT	2011	2012	2013	2014	2015	2016	2017	2018	2019
average	54.4	26.8	40.4	48.0	71.6	71.3	143.9	504.0	509.9
geom. mean	19.1	15.6	26.4	20.3	29.3	39.7	35.7	102.0	126.2
median	17	16	33	20	29	24	28	72	128

**Table 5:** Average, geometric mean and median fMRI dataset sizes (number of subjects) used in MICCAI articles in each of the years 2011–2019.

fMRI	2011	2012	2013	2014	2015	2016	2017	2018	2019
average	21.3	31.9	32.3	67.5	86.6	111.7	151.6	264.4	316.5
geom. mean	17.3	27.9	25.2	65.1	59.8	93.7	68.0	131.6	180.5
median	15	29	25	64	53	86	46	191	174

## Data Availability

All the data supporting the results was obtained from the proceedings of the 2011–2019 MICCAI conferences [13,14,15,16,17,18,19,20,21].

## References

[1] Kalayeh H.M., Landgrebe D.A. (1983). Predicting the required number of training samples. IEEE Trans. Pattern Anal. Mach. Intell..

[2] Boonyanunta N., Zaaphongsekul P. (2004). Predicting the relationship between the size of training sample and the predictive power of classifiers. Knowledge-Based Intelligent Information and Engineering Systems. KES 2004. Lecture Notes in Artificial Intelligence.

[3] Hutter M. (2021). Learning Curve Theory. arXiv.

[4] Kohli M.D., Summers R.M., Geis J.R. (2017). Medical image data and datasets in the era of machine learning—Whitepaper from the 2016 C-MIMI meeting dataset session. J. Digit. Imaging.

[5] Baro E., Degoul S., Beuscart R., Chazard E. (2015). Toward a literature driven definition of big data in healthcare. Biomed. Res. Int..

[6] Litjens G., Kooi T., Bejnordi B.H., Setio A.A.A., Ciompi F., Ghafoorian M., van der Laak J.A.W.M., van Ginneken B., Sánchez C.I. (2017). A survey on deep learning in medical image analysis. Med. Image Anal..

[7] Fukunaga K., Hayes R.A. (1989). Effects of sample size in classifier design. IEEE Trans. Pattern Anal. Mach. Intell..

[8] Adcock C.J. (1997). Sample size determination: A review. J. R. Stat. Soc. Ser. D.

[9] Eng J. (2003). Sample size estimation: How many individuals should be studied?. Radiology.

[10] Mukherjee S., Tamayo P., Rogers S., Rifkin R., Engle A., Campbell C., Golub T.B., Mesirov J.P. (2003). Estimating dataset size requirements for classifying DNA microarray data. J. Comput. Biol..

[11] Maxwell S.E., Kelley K., Rausch J.R. (2008). Sample size planning for statistical power and accuracy in parameter estimation. Annu. Rev. Psychol..

[12] Sahiner B., Pezeshk A., Hadjiiski L.M., Wang X., Drukker K., Cha K.H., Summers R.M., Giger M.L. (2018). Deep learning in medical imaging and radiation therapy. Med. Phys..

[13] Fichtinger G., Martel A., Peters T. (2011). Medical Image Computing and Computer-Assisted Intervention—MICCAI 2011, Proceedings of the 14th International Conference, Toronto, Canada, 18–22 September 2011.

[14] Ayache N., Delingette H., Goland P., Mori K. (2012). Medical Image Computing and Computer-Assisted Intervention—MICCAI 2012, Proceedings of the 15th International Conference, Nice, France, 1–5 October 2012.

[15] Mori K., Sakuma I., Sato Y., Barillot C., Navab N. (2013). Medical Image Computing and Computer-Assisted Intervention—MICCAI 2013, Proceedings of the 16th International Conference, Nagoya, Japan, 22–26 September 2013.

[16] Goland P., Hata N., Barillot C., Hornegger J., Howe R. (2014). Medical Image Computing and Computer-Assisted Intervention—MICCAI 2014, Proceedings of the 17th International Conference, Boston, MA, USA, 14–18 September 2014.

[17] Navab N., Hornegger J., Wells W.M., Frangi A.F. (2015). Medical Image Computing and Computer-Assisted Intervention—MICCAI 2015, Proceedings of the 18th International Conference, Munich, Germany, 5–9 October 2015.

[18] Ourselin S., Joskowicz L., Sabuncu M.R., Unal G., Wells W. (2016). Medical Image Computing and Computer-Assisted Intervention—MICCAI 2016, Proceedings of the 19th International Conference, Athens, Greece, 17–21 October 2016.

[19] Descoteaux M., Maier-Hein L., Franz A., Jannin P., Collins D.L., Duchesne S. (2017). Medical Image Computing and Computer Assisted Intervention—MICCAI 2017, Proceedings of the 20th International Conference, Quebec City, QC, Canada, 11–13 September 2017.

[20] Frangi A.F., Schnabel J.A., Davatzikos C., Alberola-López C., Fichtinger G. (2018). Medical Image Computing and Computer Assisted Intervention—MICCAI 2018, Proceedings of the 21st International Conference, Granada, Spain, 16–20 September 2018.

[21] Shen D., Liu T., Peters T.M., Staib L.H., Esert C., Zhuo S., Yap P.T., Khan A. (2019). Medical Image Computing and Computer Assisted Intervention—MICCAI 2019, Proceedings of the 22nd International Conference, Shenzhen, China, 13–17 October 2019.

[22] Tovino S.A. (2004). The use and disclosure of protected health information for research under the HIPAA privacy rule: Unrealized patient autonomy and burdensome government regulation. South Dak. Law Rev..

[23] Landau Y., Kiryati N. (2019). Dataset growth in medical image analysis research. arXiv.

[24] van Ginneken B., Kerkstra S., Meakin J. Grand Challenges in Biomedical Image Analysis. https://grand-challenge.org.

[25] Deng J., Dong W., Socher R., Li L., Li K., Fei-Fei L. ImageNet: A large-scale hierarchical image database. Proceedings of the IEEE Conference on Computer Vision and Pattern Recognition.

[26] Ravishankar H., Sudhakar P., Venkataramani R., Thiruvenkadam S., Annang P., Babu N., Vaidya V. (2017). Understanding the mechanisms of deep transfer learning in medical images. arXiv.

[27] Hussain Z., Gimenez F., Yi D., Rubin D. (2017). Differential data augmentation techniques for medical imaging classification tasks. AMIA Annu. Symp. Proc..

[28] Shen D., Wu G., Suk H.-I. (2017). Differential data augmentation techniques for medical imaging classification tasks. Annu. Rev. Biomed. Eng..

[29] Shin H.-C., Tenenholtz N.A., Rogers J.K., Schwarz C.G., Senjem M.L., Gunter J.L., Andriole K., Michalski M. (2018). Medical image synthesis for data augmentation and anonymization using generative adversarial networks. arXiv.

